# TBC-2, a Rab GTPase activating protein, regulates the localization of the HLH-30/TFEB and PQM-1 transcription factors in the *C. elegans* intestine

**DOI:** 10.17912/micropub.biology.002470

**Published:** 2026-10-13

**Authors:** Soumyendu Saha, Icten Meras, Christian E. Rocheleau

**Affiliations:** 1 Department of Anatomy and Cell Biology, McGill University, Montreal, QC, Canada; 2 Division of Endocrinology and Metabolism, Department of Medicine, McGill University, Montreal, QC, Canada; 3 Metabolic Disorders and Complications Program, RI-MUHC, Montreal, QC, CA

## Abstract

Insulin/IGF signaling (IIS) inhibits the nuclear localization of the DAF-16 /FOXO transcription factor to regulate longevity and stress resistance in *C. elegans .* In the intestine, IIS promotes DAF-16 localization to endosomes and loss of TBC-2 , a RAB-5 GAP, results in increased endomembrane localization of DAF-16 at the expense of nuclear localization, decreased DAF-16 target gene expression, longevity and stress resistance. Here we found that TBC-2 differentially regulates the localization of the IIS-regulated transcription factors PQM-1 and HLH-30 /TFEB. Our results suggest a broader role for TBC-2 in regulating IIS and that TBC-2 likely functions at an upstream point in the IIS pathway.

**Figure 1. TBC-2 regulates the localization of PQM-1 and HLH-30:** (A-A') Representative confocal images of PQM-1::GFP in the intestine of wild type and *tbc-2(tm2241)* mutants showing nuclear (n) and endomembrane localization (yellow arrow). ^ denotes autofluorescent gut granules. (B, C) Bar graphs showing the percent animals with PQM-1::GFP localization to endomembranes (B) and the nucleus (C). Experimental triplicates with 30-50 animals per replicate. (D) Box and scatter graph representing the ratio of nuclear to cytosolic fluorescence intensity of PQM-1::GFP in wild type ( *n* =26) and *tbc-2(tm2241)* mutants ( *n* =29), each data point is an individual intestinal cell. (E-E'') Confocal images of endogenously tagged HLH-30::3xFlag::eGFP in the intestine of wild type with and without endomembrane localization (E-E') and in *tbc-2(tm2241)* (E''). Nuclear (n) and endomembrane localization (yellow arrows). (F) Bar graph showing the percent animals with endomembrane localization of HLH-30::3xFlag::eGFP, HLH-30::GFP, and HLH-30::RFP in wild type and *tbc-2(tm2241)* mutants. Experimental replicates with 19-36 HLH-30::3xFlag::eGFP animals per replicate and 30-60 animals per replicate for HLH-30::GFP and HLH-30::RFP. (G) Box and scatter graphs representing the ratio of nuclear to cytosolic fluorescence intensity of HLH-30::3xFlag::eGFP, HLH-30::GFP, and HLH-30::RFP in wild type and *tbc-2(tm2241)* mutants, *n* > 18 for each condition *.* (H) Bar graph showing the fold change in *lgg-1* mRNA expression levels between wild type and *tbc-2(tm2241)* mutant, in 3 biological replicates. (I-I'') Representative confocal images DAF-16::GFP and HLH-30::RFP colocalization in *tbc-2(tm2241)* as shown by the yellow arrow. ^ marks the auto fluorescent gut granules and n denotes nuclei. (J) Bar graph showing the percent animals with endosomal HLH-30::GFP in the *tbc-2(tm2241)* background, fed control (ev, empty vector) RNAi or RNAi targeting components of the IIS pathway. Each replicate represents >25 worms per condition. Paired Student T-test was used to analyse data in (B, C, F, H and J) and unpaired Student T-test was used to analyse data in (D and G). Scale bar, 10 μm. *ns* , not significant, * *P < 0.05* , ** *P<0.01* and **** *P<0.0001* .

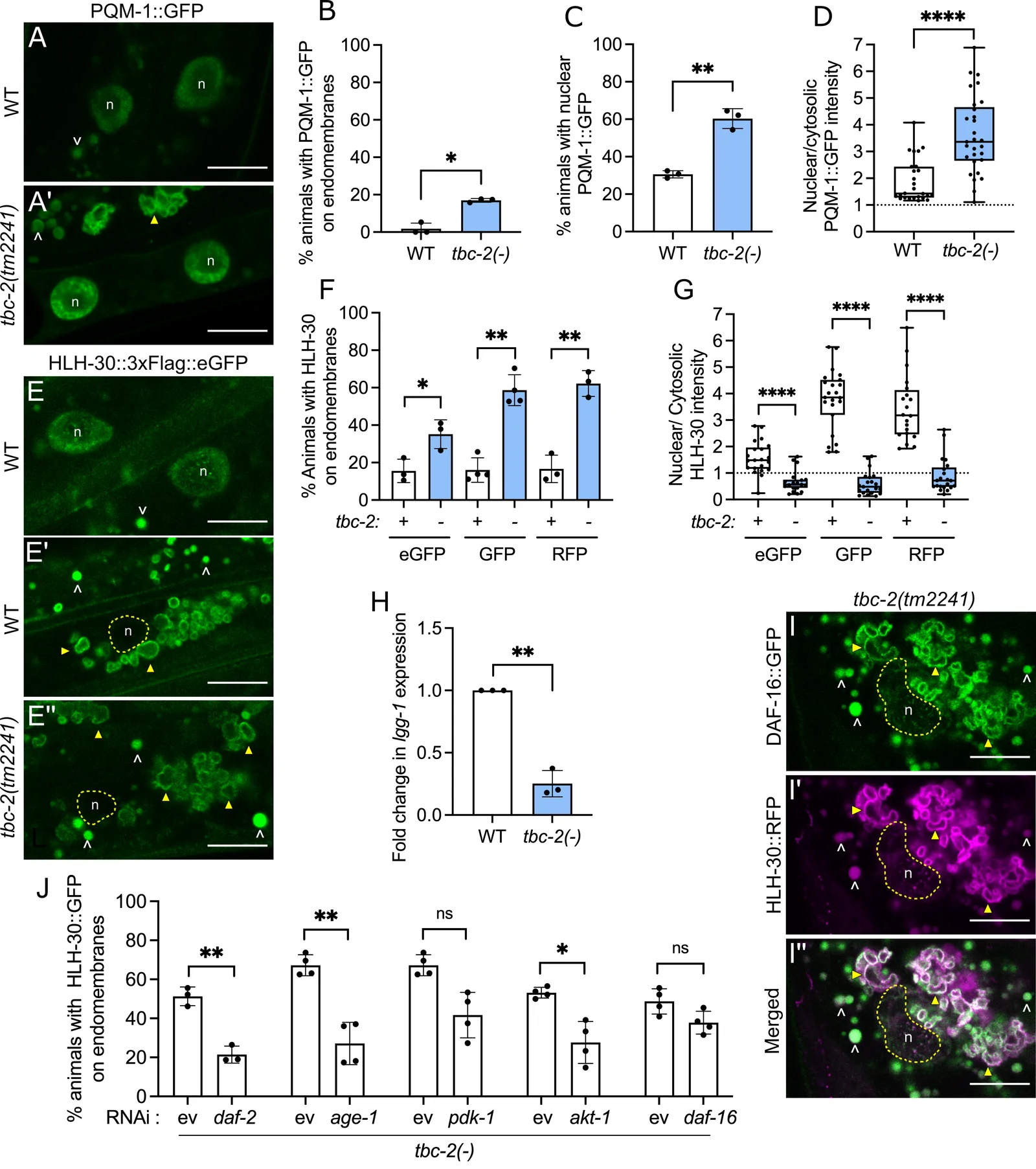



## Description

Insulin/Insulin-like Growth Factor (IGF) signaling (IIS) is an evolutionarily conserved regulator of metabolism (Barbieri et al., 2003; Murphy & Hu, 2013). In *C. elegans* , the IIS pathway regulates lifespan, fat storage and resistance to various stresses (Friedman & Johnson, 1988; Kenyon et al., 1993; Kimura et al., 1997; Lithgow et al., 1994; Murphy & Hu, 2013; Ogg et al., 1997). The core pathway is defined by the DAF-2 insulin/IGF receptor, the AGE-1 class I PI3 kinase which generates PI(3,4,5)P which in turn recruits PDK-1 and AKT-1 /2 kinases to the plasma membrane where PDK-1 phosphorylates and activates AKT-1 /2. A major target of *C. elegans* IIS is the inhibition of the DAF-16 /FOXO transcription (Kimura et al., 1997; Lin et al., 1997; Morris et al., 1996; Ogg et al., 1997; Paradis et al., 1999; Paradis & Ruvkun, 1998). Phosphorylation of FOXO transcription factors by AKT kinases results in cytoplasmic sequestration (Brunet et al., 1999).

The *C. elegans* intestine, while only 20 cells, is a major metabolic organ and site of many IIS-mediated functions (McGhee, 2007; Murphy & Hu, 2013; Zhang et al., 2022). We previously demonstrated that DAF-16 localizes to endosomes in the intestinal cells (Meras et al., 2022). Interestingly, DAF-16 positive endosomes are not present in every cell or every animal and can vary from just a few to hundreds. We found that TBC-2 regulates DAF-16 localization. TBC-2 is a RAB-5 GTPase Activating Protein (GAP) that regulates early to late endosome maturation (Chotard et al., 2010). In *tbc-2 ( tm2241 )* deletion mutants, DAF-16 localizes to irregularly shaped endomembranes to a greater extent than wild type and at the expense of nuclear localization (Meras et al., 2022). As such, TBC-2 antagonizes IIS-mediated gene expression, longevity, fat storage and resistance to several stresses (Meras et al., 2022; Traa et al., 2023). To determine if this localization was specific to DAF-16 we looked at two additional transcription factors that are differentially regulated by IIS, PQM-1 and HLH-30 .

PQM-1 is a zinc finger transcription factor required for the long lifespan of *daf-2 (-)* mutants (Tepper et al., 2013). Localization of PQM-1 is opposite that of DAF-16 whereby IIS promotes nuclear localization of PQM-1 while it antagonizes DAF-16 (Tepper et al., 2013). In *tbc-2 (-)* animals, PQM-1 ::GFP showed some localization to endomembranes ( Figure 1A- A', B). Despite the endomembrane localization of PQM-1 , there was a significant increase in the number of animals with nuclear localized PQM-1 and an increase in the nuclear to cytosolic fluorescence intensity ratio of PQM-1 in *tbc-2 (-)* mutants as compared to wild type ( Figure 1C, D). This is consistent with the antagonistic genetic relation between PQM-1 with DAF-16 localization (Tepper et al., 2013).

HLH-30 or TFEB (Transcription factor EB) is a transcription factor involved in lysosomal biogenesis and acts in a combinatorial manner with DAF-16 in response to the starvation stress (Lapierre et al., 2013; Lin et al., 2018; O'Rourke & Ruvkun, 2013). In wild type we observed that endogenously tagged HLH-30 ::3xFlag::eGFP was mostly nuclear with some cytoplasmic and endomembrane localization ( Figure 1E- E', F). In *tbc-2 (-)* mutants, we observed an increase in the number of animals with HLH-30 ::3xFlag::eGFP localized to endomembranes ( Figure 1E '', F). Similar results were seen with HLH-30 ::GFP and HLH-30 ::RFP transgenic animals ( Figure 1F ). Like DAF-16 , HLH-30 showed reduced nuclear to cytosolic fluorescence intensity in *tbc-2 (-)* mutant animals ( Figure 1G ). To test if the reduced nuclear localization of HLH-30 affected downstream HLH-30 dependent gene expression, we analysed the mRNA expression of *lgg-1* . LGG-1 is ATG8 in yeast and LC3 in mammals and is responsible for autophagosome formation (Melendez et al., 2003). LGG-1 expression is dependent on HLH-30 , such that *lgg-1* mRNA levels are suppressed in *hlh-30 (RNAi)* (Lapierre et al., 2013) *.* There was a significant decrease in the *lgg-1* mRNA levels in *tbc-2 (-)* mutants consistent with the reduced nuclear localization of HLH-30 ( Figure 1H ).

We observed that DAF-16 and HLH-30 co-localized on endomembranes in *tbc-2 (-)* mutants indicating that TBC-2 might regulate these transcription factors by a common underlying mechanism ( Figure 1 I-I''). To test if IIS regulates HLH-30 localization on endosomes as seen with DAF-16 (Meras et al., 2022), we use RNAi to systematically knock down components of the IIS pathway- DAF-2 , AGE-1 , PDK-1 , AKT-1 and DAF-16 in *tbc-2 (-);* HLH-30 ::GFP animals. Compared to empty vector control, RNAi mediated knock-down of *daf-2 , age-1* and *akt-1* significantly decreased the number of *tbc-2 (-)* mutant animals with HLH-30 on endomembranes ( Figure 1J ). *pdk-1 (RNAi)* significantly suppressed HLH-30 endomembrane localization in three of four experiments ( Figure 1J ). *daf-16* RNAi did not decrease the endomembrane localization of HLH-30 ( Figure 1J ). Thus, IIS signaling promotes the localization of HLH-30 to endomembrane compartments as it does with DAF-16 .

In conclusion, this study builds upon our previous findings that TBC-2 regulates DAF-16 localization to endomembranes (Meras et al., 2022). We demonstrated that TBC-2 regulates the localization of two additional IIS regulated transcription factors, HLH-30 and PQM-1 , further supporting a role for TBC-2 in antagonizing IIS in the intestine. Similar to DAF-16 , we found that HLH-30 localizes to uncharacterized endomembrane compartments in *tbc-2 (-)* mutants at the expense of its nuclear localization. The reduced expression of the HLH-30 target, *lgg-1* , in *tbc-2 (-)* mutants suggests that this impacts HLH-30 transcriptional activity.

We found that HLH-30 colocalizes with DAF-16 on the same endomembranes in *tbc-2 (-)* mutants suggesting a similar mode of regulation. HLH-30 /TFEB and DAF-16 /FOXO are inhibited by phosphorylation and binding with 14-3-3 proteins that sequester them in the cytoplasm (Brunet et al., 1999; Colino-Lage et al., 2024; Li et al., 2007; Lin et al., 2018; Martina et al., 2012; Roczniak-Ferguson et al., 2012). We previously demonstrated that FTT-2 /14-3-3 and DAF-16 phosphorylation regulate DAF-16 localization to endomembranes (Meras et al., 2022). We anticipate that HLH-30 is also sequestered in a similar manner. However, we don't know whether 14-3-3 mediates HLH-30 and DAF-16 binding to these endomembrane structures or if there are additional factors involved.

DAF-16 and HLH-30 forms a complex and regulates overlapping stress response genes and lysosome morphology (Lin et al., 2018; Ricaurte-Perez et al., 2025). Whether they form a complex on endomembranes remains unclear. RNAi of *daf-16* did not affect the localization of HLH-30 to endomembranes suggesting that there may not be a codependence for endomembrane localization. However, this should be further tested with null alleles.

Like DAF-16 , we also found that IIS partially regulates the localization of HLH-30 to endomembranes. Thus, it is possible that other metabolic or stress response pathways such as AMPK, Tor, or JNK could be involved (El-Houjeiri et al., 2019; Greer et al., 2007; Lapierre et al., 2013; Martina et al., 2012; Oh et al., 2005; Roczniak-Ferguson et al., 2012; Settembre et al., 2012). Unlike DAF-16 and HLH-30 , IIS promotes nuclear localization of PQM-1 (Tepper et al., 2013). Although we detected some endomembrane localization of PQM-1 in *tbc-2 (-)* mutants, we found that the nuclear versus cytoplasmic localization of PQM-1 was increased suggesting increased nuclear import or possibly an increase in PQM-1 levels. This is consistent with our previous finding that *tbc-2 (-)* mutants had increased expression of *vha-6* , a top PQM-1 target (Meras et al., 2022; Tepper et al., 2013). Taken together these results suggest that TBC-2 antagonizes IIS at a common point upstream of the DAF-16 , HLH-30 and PQM-1 transcription factors.

## Methods

All strains were derived from the wild type N2 strain. Worms were maintained at 20°C on NGM plates as described in WormBook (www.wormbook.org). The strains were fed HB101 *E. coli* as a food source.

For RNAi feeding experiments fourth larval stage (L4) worms were placed on RNAi plates and transferred to fresh plates each day for 3 days. L4 progeny from the day 3 plates were scored for the experiment. RNAi feeding clones, except *pdk-1* , were obtained from the Ahringer RNAi library (Fraser et al., 2000; Kamath et al., 2000; Kamath et al. 2003). *pdk-1* RNAi clone (pQcSS01) was generated by PCR of worm lysates using primers SSA38 5' - CAACTAAGATCTATTTCGTGATCGGACTTGTTG – 3' and SSA39 5' – GTCATACTCGAGGCTAGCATCATTTCCCAAATTCATC -3' and ligated to the L4440 vector from Addgene (https://www.addgene.org/1654/).

Live imaging of the L4 hermaphrodite worms was done at room temperature. The worms were transferred to a 2% agarose pad and immobilized with 10 mM levamisole. They were scored for nuclear or endosome localization of PQM-1 , DAF-16 and HLH-30 under the fluorescent ZeissA1 axio Imager. To measure the fluorescence intensity of nuclear PQM-1 and HLH-30 L4 worms were imaged using a Zeiss Axio Observer Z1 LSM780 laser scanning confocal microscope in the Molecular Imaging Platform of the RI-MUHC. Image analysis was performed using Fiji (ImageJ).

For quantitative RT-PCR, RNA was extracted from young adult *C. elegans* using TRIZOL as explained in (Meras et al., 2022). LGG-1 was amplified using forward primer 5' – ACCCAGACCGTATTCCAGTG -3' and reverse primer 5' – ACGAAGTTGGATGCGTTTTC -3'.

## Reagents

**Table:** 

**Strain**	**Genotype**	**Source**
OP201	*unc-119 ( tm4063 ) III; wgIs201 [ pqm-1 ::TY1::EGFP::3xFLAG(92C12) + unc-119 (+)] *	CGC; (Sarov et al., 2006; Zhong et al., 2010)
QR1112	*tbc-2 ( tm2241 ) II; wgIs201 [ pqm-1 ::TY1::EGFP::3xFLAG(92C12 + unc-119 (+)] *	This study
VZ892	*hlh-30 ( syb1452 [ hlh-30 ::3xFlag::eGFP]) IV *	CGC; (Martina et al., 2021)
QR1138	*tbc-2 ( tm2241 ) II; hlh-30 ( syb1452 [ hlh-30 ::3xFlag::eGFP]) IV *	This study
JIN1679	*jinEx10 [ hlh-30 p:: hlh-30 ::GFP + rol-6 ( su1006 )] *	CGC; (Lapierre et al., 2013)
QR1068	*tbc-2 ( tm2241 ) II; jinEx10 [ hlh-30 p:: hlh-30 ::GFP + rol-6 ( su1006 )] *	This study
WU1598	*hlh-30 ( tm1978 ) IV; hlh-30 p:: HLH-30 ::RFP + rol-6 ( su1006 ) *	(Murphy et al., 2019)
QR1084	*tbc-2 ( tm2241 ) II; hlh-30 p:: HLH-30 ::RFP + rol-6 ( su1006 ) *	This study
QR1101	*daf-16 ( hq23 [ DAF-16 ::GFP]) I; tbc-2 ( tm2241 ) II;; hlh-30 p:: HLH-30 ::RFP+ rol-6 ( su1006 ) *	This study
		

**Table:** 

**RNAi clone**	**Gene**	**Source**
L4440	Empty vector control	(Timmons & Fire, 1998)
III-7G12	*daf-2*	(Kamath et al., 2003)
II-7J02	*age-1*	(Kamath et al., 2003)
V-7I17	*akt-1*	(Kamath et al., 2003)
I-5M24	*daf-16*	(Fraser et al., 2000)
pQcSS01	*pdk-1*	This study
